# Diverse Radiofrequency Sensitivity and Radiofrequency Effects of Mobile or Cordless Phone near Fields Exposure in *Drosophila melanogaster*

**DOI:** 10.1371/journal.pone.0112139

**Published:** 2014-11-17

**Authors:** Styliani Geronikolou, Stelios Zimeras, Constantinos H. Davos, Ioannis Michalopoulos, Stephanos Tsitomeneas

**Affiliations:** 1 Biomedical Research Foundation of Academy of Athens, 4 Soranou Efessiou Str, 11527, Athens, Greece; 2 Department of Electronics, TEI of Piraeus, Petrou Ralli & Thivon 250, 122 44, Athens, Greece; 3 Department of Mathematics, Division of Statistics and Actuarial-Financial Mathematics, University of the Aegean, 82300, Karlovassi, Samos, Greece; Ecole Normale Supérieure, France

## Abstract

**Introduction:**

The impact of electromagnetic fields on health is of increasing scientific interest. The aim of this study was to examine how the *Drosophila melanogaster* animal model is affected when exposed to portable or mobile phone fields.

**Methods/Results:**

Two experiments have been designed and performed in the same laboratory conditions. Insect cultures were exposed to the near field of a 2G mobile phone (the GSM 2G networks support and complement in parallel the 3G wide band or in other words the transmission of information via voice signals is served by the 2G technology in both mobile phones generations) and a 1880 MHz cordless phone both digitally modulated by human voice. Comparison with advanced statistics of the egg laying of the second generation exposed and non-exposed cultures showed limited statistical significance for the cordless phone exposed culture and statistical significance for the 900 MHz exposed insects. We calculated by physics, simulated and illustrated in three dimensional figures the calculated near fields of radiation inside the experimenting vials and their difference. Comparison of the power of the two fields showed that the difference between them becomes null when the experimental cylinder radius and the height of the antenna increase.

**Conclusions/Significance:**

Our results suggest a possible radiofrequency sensitivity difference in insects which may be due to the distance from the antenna or to unexplored intimate factors. Comparing the near fields of the two frequencies bands, we see similar not identical geometry in length and height from the antenna and that lower frequencies tend to drive to increased radiofrequency effects.

## Introduction

There is an increasing public and scientific interest on studying the impact of natural (i.e. solar) and artificial electromagnetic fields on health. The electric power devices, base stations, cell phones, mobile phones, portable devices (i.e. wifi routers, cordless phones) are part of our daily life, transmitting non-ionizing radiation. The effects of this radiation on health that have been published in scientific literature vary upon the experimental design, the epidemiological focus and the given research conditions. These effects are generally divided in two categories: thermal and non thermal, although subtle thermal effects have been suggested as a third level of effects which lies in between [1].

Various non-thermal biological effects on human or animal models have been reported: Ca^++^ ions efflux in cellular membrane, hormonal (melatonin, serotonin, TSH, sex hormones) level decrease, DNA linkage, blood pressure increase, etc [2]–[7]. All known studies have used different epidemiological, behavioural, technical and experimental methodologies in order to cover all aspects of biological effects. However, most of the epidemiological studies were retrospective and were subjected to biased scientific criteria [1]. The European Commission in its 2007 Scientific Committee on Emerging and newly identified risks (SCENIHR) report [8], refer to Foster and Repacholi (2000) review which had concluded that: ‘attempts at environmental analysis of the effects of environmental EMF, with few exceptions have been scattered in focus, sporadic in publication and uneven in quality’ [9]. All epidemiological studies had presumed that the electromagnetic emission was stable and consistent throughout the experiments, but this is challenged by technical reports [10]. The *in vivo* laboratory studies related to radiation consequences, involved various experimental models such as *Drosophila melanogaster, Saccharomyces, Rattus, Mus musculus* etc. In the SCENIHR report mentioned above, the electromagnetic sensitivity of insects has been recognised as likely to be particularly sensitive to electromagnetic fields.

The *Drosophila* animal model has been thoroughly used to study non thermal consequences and proved to be a relevant biomarker of value [11]. The egg laying of sexually mature flies, males and females, coming from pupae and radiated for several minutes in microwave frequencies was studied [12]. This kind of exposure produces confusing results as these frequencies can augment the body temperature of the model. Another study excluded the male insects from radiation, whereas the females were already impregnated before exposure [13]. This had been a rather risky and complicated effort with non credible results, as the mortality of eggs or larvae coming from the 4 days old insects, was high. A population exposed to high temperature (37°C) and radiation was compared to a population exposed to high temperature only by counting the oviposition (number of laid eggs) of the total insect population [14]. Oregon R type of *Drosophila* cultures were exposed continuously and non-continuously to 900 MHz and 1880 mHz driving to a decrease of the insects productivity in a statistically significant level [15]–[19]. Most of the aforementioned experiments report many non-developed eggs in both control and exposed populations besides reduced oviposition. In addition, all of them arbitrarily presumed that the statistical distributions of the egg laying were normal.

The aim of our study was to investigate the effect of the 900 MHz and 1880 MHz near fields electromagnetic emission on *Drosophila melanogaster* oviposition in a way to overcome the above mentioned systematic errors.

## Materials and Methods

### Animal Model

The animal model of choice for the radiation impact query has been the wild type Oregon R of *Drosophila melanogaster* (from stock), an insect belonging to *Diptera*. This is a frequently studied animal model offering advantages as: short life time cycle, 54–70% homologous alleles with the human genome [19]–[22], and a process timing of its metamorphic stages to developmental ones [23]. Additionally, the *Drosophila* is a unique and ideal model organism for conducting neuro-endocrine genomics research because, unlike other models, it has adipose-like tissues and a lipid transport system, making it a closer model to humans. Drosophila oogenesis system seems to be befitting for the investigation of electromagnetic field potential impact on biological settings [11].

### Food and culture

The flies were kept in 50 ml glass vial with 10 cm height and 2.5 cm diameter, taped with cotton plug and were incubated at 25°C, with 12 hours periods of light and darkness. The relative humidity of the room was measured at 70%. All glass vials had been sterilised in a dry air oven for 90 min at 160°C, or in moist heat sterilization for 35 min before the food preparation.

The food for the cultivation consisted of: 4 g agar, 13 g yeast, 16 g sugar, 25 g tomato pulp, 32 g rice flour, 2 ml propionic acid and 2 ml ethanol diluted in 450 ml distilled water. This mixture was sterilised by a 10 min boiling procedure. 2 ml of propionic acid and equal quantity of ethanol were added in order to ensure safe preservation conditions. The food had been prepared and thickened in room temperature conditions before being added into sterilised glass vials. The food formed an 1 cm thick smooth surface at the bottom of the each vial. Finally, all the glass vials with food were kept at 40°C. In order to enhance insect oviposition, 2–3 drops of thick water- diluted yeast had been added, 12–16 hours before the experimental procedure.

### Experimental procedure

The experiments to be presented herein, have taken place in the University of Athens Biology Quarters. Each experiment included a collection of newly emerged flies from the stock. The newly emerged insects were anaesthetised with ether and separated under stereoscopic microscopy (Carl Zeiss 4773117) into sex groups. Male and female insects were placed in different glass vials with food at 25°C. Each vial was then exposed continuously for 20 min every day for two days, until insects were sexually mature. The mature insects were anaesthetised again and placed in new glass vials with food; each vial contained 8 male and 8 female insects. These new cultures were exposed for the same time period for three more days. Six days after the last day of radiation we measured the number of chrysalides on the vial wall. Three days later we counted the newly emerged insects as introduced by Panagopoulos et al 2004 [18], The newly emerged flies (pupae) encounter low mortality during their transformation risk in larvae [23].

We used two different electromagnetic sources for the cultures to be exposed: the cordless phones emitting frequency technically reported as 1880 MHz and the mobile phone (Global System for Mobile Telecommunications) frequency spectrum at 864.1 and 868.1 MHz emitted by common mobile phones.

**Figure 1:**
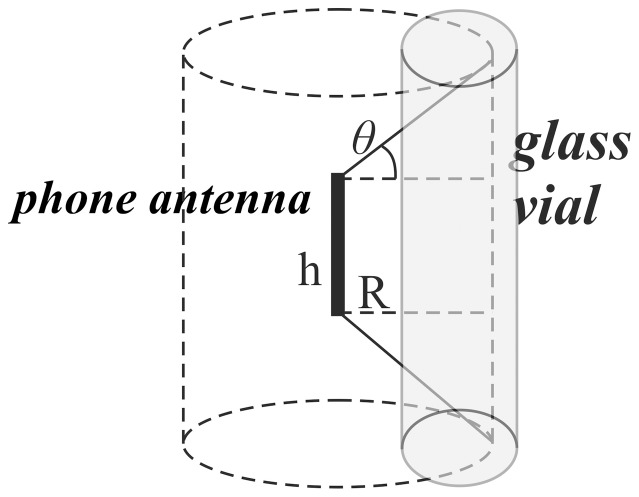
Experimental exposure graphic.

We kept the field characteristics same as in typical use (under the ICNERP limits) during speaking and we have placed the phone device in contact with the glass vial during the experimenting time. Thus, we exposed the cultures to the complicated and intense near field which is extended a couple of centimetres from the radiation antenna. This procedure was repeated five times for the cordless phones (repetitions 1–5) and five times for the second generation mobile phones (repetitions 6–10). The non exposed cultures (control group) were kept away from any electromagnetic source under the same room and temperature conditions. The experiment is graphically described in Figure 1.

The flies were placed in the vicinity of the antenna of the mobile and cordless phones. Theoretical calculations by physics were made for the estimation of each exposure which depends on the frequency, power output, antenna configuration and on the induction and radiation field. This leads to a very complicated electric E_EXP_ and magnetic H_EXP_ field exposure with increased uncertainty. In such cases, the average exposure depends dominantly on the non-irradiated fields E(1/R^2^), E(1/R^3^) and H(1/R^2^), decreased by the square or cube of the distance, and not so much on the radiation field E(1/R) and H(1/R), which is decreased by distance. The intensity ratio of the non-radiated to the radiated fields, resulting from the equations of the electromagnetic field, and assuming that the distance R is obtained in the middle of the antenna and it is perpendicular to it, is as follows:



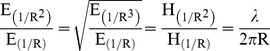



Because the experiment took place very close to the phone, its near field is more intense than its far (radiated) field. These findings may lead to a general exposure prediction, with some obvious assumptions, based on the type of portable phone, on the exposure distance R and on the type of exposed tissues. A simple estimation can be made by assuming cylindrical flow of the power output, and transmitter electromagnetic power output P which spreads in the lateral area A of a cylinder surrounding the phone antenna. The cylinder has a radius R and height equal to the effective height h of the antenna plus the factor 2Rtanθ, with the angle θ corresponding to the antenna radiation lobe. In this case, we use the relations:



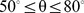


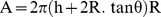


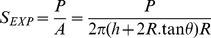



Applying these relations to the phones used, with the assumption of the active antenna size h≅0.16λ and the maximum power output P = 2 W (cellular phone) and P = 0.25 W (cordless phone), we estimated the approximate exposure, inside the glass vial.

**Figure 2:**
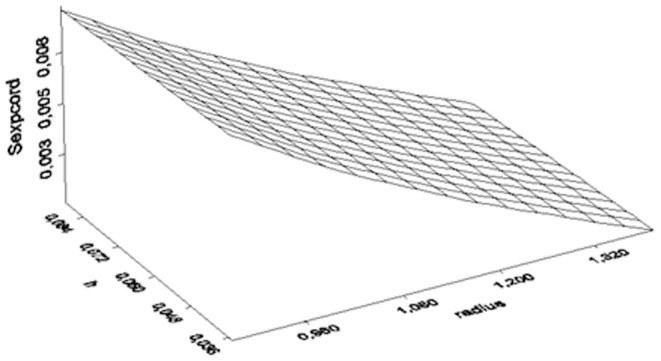
Three dimensional illustration of the Sexp for 1880 MHz vs h and radius.

**Figure 3:**
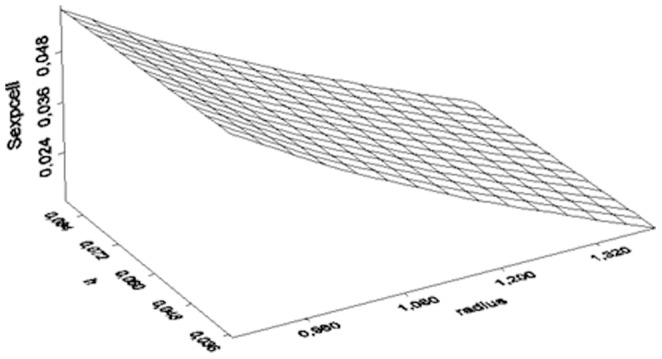
Three dimensional illustration of the Sexp for 900 MHz vs h and radius.

Figures 2 and 3 illustrate the three dimensional representation of the S_exp_ function for 1880 MHz and 900 MHz for different h and radius for the ease of representation.

When the antenna size (h) is small and the cylinder radius (R) is small the S_exp_ is strong - as both measures (h and R) increase the S_exp_ becomes weaker (close to zero).

**Figure 4:**
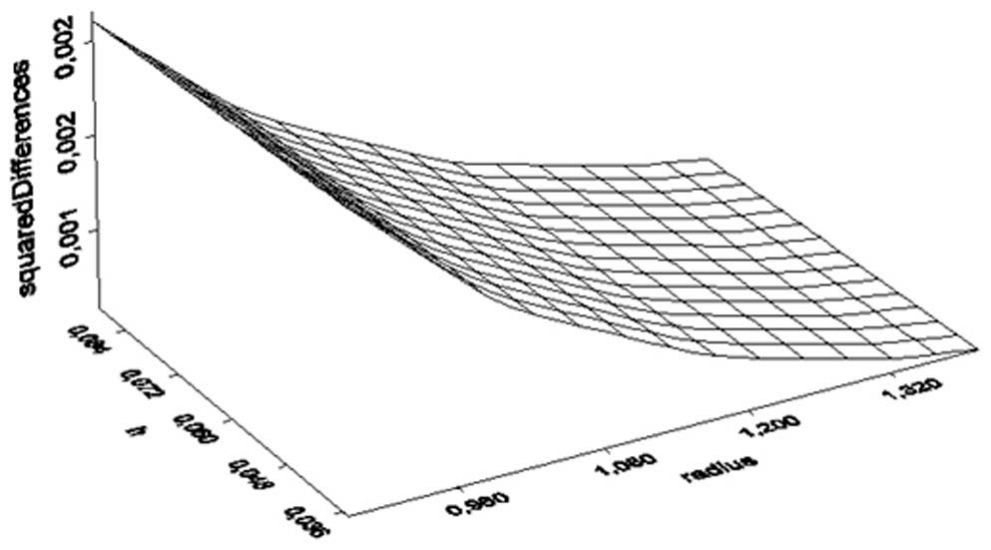
Three dimensional illustration of the squared differences between 1880 and 900 MHz vs h and radius.

In order to compare the two electromagnetic powers (1880 and 900 MHz), a variable D_Sexp_ is defined according to following equation:
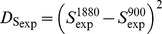



Comparison of the two electromagnetic powers (1880 and 900 MHz) suggests that when the antenna size (h) is small and the cylinder radius is small the D_Sexp_ is large (indication of differences). As both measures (h and radius) increase the D_Sexp_ becomes negligible (no differences) (Figure 4).

The Specific Absorption Rate had been approximately determined as 0,08 W/kg for the cordless phone [24]–, and 0,67 W/kg for the mobile phone.

The Specific Absorption Rate (SAR) of our fruit flies model was not measured, but calculated, whereas we used the values σ (electric field conductivity) and ρ (electric field intensity) proposed by Lee in 2008 [26]: σ = 1,19 and ρ = 1000 kg/m^3^ for flies ([26]. Furthermore, in estimating the value of SAR via the formula:
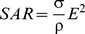



one needs a reliable estimate of the value of the electric field E (electric power in Volts/meter). As 
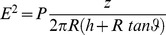


It is well known that in the near field limit z (impendance of free space 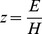) oscillates very quickly in space, so that for a fixed position its instantaneous value can practically vary from zero to infinity and can change very quickly from one measurement to another. This implies that a proper analysis requires that both E and H be measured by detailed in space and time near field measurements. However, in our experiment such measurements are practically impossible to perform in a meaningful way, since flies have a quite small size rendering space averaged measurements of no practical use. Instead, we naively, implemented the OSHA protocol which is properly applicable only to the far field limit, in order to obtain an indicative estimate of the required values. We, thus, use the formula:
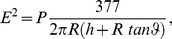



where 377 is a fixed constant equal to the ratio E to H (magnetic strength in Amperes/meter) or the characteristic impedance of free space.

Results based on the above formula can only be considered as order of magnitude estimates: for the calculated cordless phone is calculated SAR_1880_ = 7*10^−4^ W/kg, and the SAR value for the mobile phone is calculated SAR_900_ = 5*10^−3^ W/kg for the flies.

Animal subjects were exposed to radiofrequencies with intensity below existing safety limits.

### Statistical Analysis

Data are expressed as mean values ± SD or proportions. Albeit Shapiro Wilks resulted to normality of all distributions, their size is so small that we had to assume that the distributions were unknown. As a result, group means were compared with the use of Mann-Whitney non parametrical test. All p-values are two-sided with a value of p<0.05 considered as statistically significant. Statistical analyses were performed with SPSS 17 software. To overcome the situation with the small samples and measure the effect size of the observed difference between these small samples we applied the modification proposed by Lipsey & Wilson (2001) [27] and Durlak (2009) [28] on Hedge's g statistics formula (Hedges' g is a variation of Cohen's d that corrects the bias due to small sample sizes) [29]: 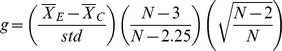
where 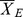 and 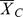 are the mean values of the experimental and the control groups alternatively, std is the standard deviation given by the formula:
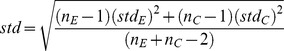



where *n_E_* and *n_C_* are the number of the experimental and the control groups, *std_E_* and *std_C_* are the standard deviations of the experimental and the control groups, and N = *n_E_*+*n_C_*.

## Results

In the first five sets of experiments, we separated the insects into two groups: a) the Exposed 1880 MHz cordless phone group and b) the Unexposed group. In the second five set of experiments we separated the insects into two groups: c) the Exposed to 900 MHz mobile phone group and d) the unexposed group.

For the cordless phone 1880 MHz field the mean for the Ovicity in vials (corresponding to the number of laid eggs per maternal fly) was 10.58±1.45 and the relevant mean for the control was 12.45±0.384 (Table 1). The Mann Whitney test showed a limited statistical difference in the fecundity estimates of pupae between radiated by 1880 MHz and not radiated group z = 2.209, p = 0.0445 with an effect size g = 1.42. The mean ovicity in the experimental repetitions 6–10 with a mobile phone was 8.52±0.60, whereas, the mean ovicity of the unexposed cultures was 12.42±0.794 (Table 2). The Mann Whitney test showed a significant statistical difference between control culture and mobile phone radiated culture z = −2.611, p = 0.009 with an effect size g = 4.43.

**Table 1:** Experimental results from cultures exposed to near field of a cordless phone (1880 MHz) p = 0.0445 between control and cordless phone radiated cultures.

Experiment Nr	Culture radiated with cordless phone Mean Ovicity in 20 vials (egg nr ratio)	Control Mean Ovicity in 20 vials (egg nr ratio)
1	9.7	12.25
2	9.6	13.1
3	12.4	12.4
4	11.9	12.1
5	9.3	12.4
Sum	52.9	62.25
Mean	10.58±1.45	12.45±0.384

**Table 2:** Experimental results from cultures exposed to near field of a second generation mobile phone (900 MHz) p = 0.0090 between control and mobile phone radiated cultures.

Experiment Nr	Culture radiated with mobile phone Mean Ovicity in 20 vials (egg nr ratio)	Control Mean Ovicity in 20 vials (egg nr ratio)
6	9.05	13.5
7	7.8	11.4
8	7.95	12.4
9	9.1	12.8
10	8.7	12
Sum	42.6	62.1
Mean	8.52±0.60	12.42±0.794

The Mann Whitney test for the difference between the oovicity in the mobile phone 900 MHz culture and 1880 MHz mobile phone field culture results in a statistical significant difference between cordless phone and mobile phone exposed groups (z = −2.6 p = 0.008, with an effect size g = 1.48) but not a significant difference between the two unexposed groups (z = −0.106, p = 0.916, with an effect size g = 0.039).

As far as the correlation analysis is concerned (Spearman correlation coefficient varies from −1 to +1), strong correlations were found between the control for 1880 MHz mobile phone field and the control for 900 MHz mobile phone field (r = −0.872, p = 0.054) as well as between the insects exposed to 900 MHz mobile phone field and its control (r = 0.800, p = 0.104).

## Discussion

In our study, we questioned the instrumental assessment of electromagnetic fields characteristics in such short distance from the emitting mobile telephone antenna, as they provide very poor accuracy. Furthermore, insects body mass is much lower than humans and, thus, an instrumental (point) measurement is not even indicative of the field that each insect is exposed as the field shifts rapidly. Consequently, Specific Absorbance rate is also difficult if not impossible to be calculated, but in order of magnitude. At 900 Hz for example it is well known that the near field wavelength extends to 0.053 m [25], [30]–[32].

Direct measurement of the exposure of each subject is not suggested in environmental epidemiology, because problems related to environmental statistics may arise [32]. In the same time, the electric E_EXP_ and magnetic H_EXP_ field exposure is highly complicated with increased uncertainty in such narrow antenna-subjects vicinity. Thus, we negated the instrumental measurements and calculated by physics the two types of phones near fields inside the experimenting vials. Followingly, we simulated these near fields and represented them in three dimensionsional figures in different but informative scales (z-axis) in Figures 2 and 3 and we simulated and represented in a three dimensional figure the difference of these near fields also (Figure 4).

For environmental monitoring, researchers use different statistical models to assign exposures to individuals. The interpretation of these models may result in complicated errors in the health effect estimates if the distribution of the radiation field is arbitrarily treated as known. This has been a systematic error in most previous epidemiological and experimental assays related to the electromagnetic field impact on health. This issue is known but still unsolved [1]. This exposure error is neither “classical” nor “Berkson” [33], so standard regression calibration methods could not apply [33]–[35]. To overcome this problem we assumed that all distributions were unknown. This assumption can release our statistical model from implicating linear approaches and complex systematic errors [35].

This non parametrical model is flexible enough to include a wide range of common situations, but at the same time allow most of the familiar ideas of normal linear regression to carry over. Furthermore, we measured the effect size of each observation with g measure- modified by Lipsey and Wilson [27] and Durlak [28] based on the bias correction for small samples introduced by Hedge's g [29].

Unlike other research studies in literature [6], [16], our analysis showed that the radiation with cordless phones does not arrive to unquestionable guilt of the stressful agent due to the limited statistical significance shown. This means that either one or more other environmental factors (i.e. room temperature or humidity) or one or more intimate factors affected the insects' fecundity. Given the fact that, both experiments were performed under the same and rather stable microclimatic circumstances, this limited statistical result implies either an intimate factor (i.e. genetic polymorphism, diverse radiofrequency sensitivity) or designates either the studied frequency of exposure, or the the intensity or weakness of the near field based on the fly's vicinity to the antenna during assessment (Fig 1, 2, 3, 4). In other words, the frequency of 1880 MHz might represent a threshold frequency under which possible biological effects may happen and additionally, each subject taking part to each experimental repetition may react differently in electromagnetic stress (diverse radiofrequency sensitivity i.e. due to genetic factors). Moreover, the difference in response to each frequency stimulation is validated by our results that bring to light that lower frequency (900 MHz herein) is a more intense stress factor than 1880 MHz (g_900_>g_1880_). Although significant decrease in Drosophila melanogaster fecundity after mobile phone exposure has been observed before [11], [16], [18], [36], in our opinion it is the first time that the effect is validated, simulated, measured and given in order of magnitude and/or of the near field inhomogeneity. The two near fields effects are compared with known advanced and sophisticated statistical techniques bringing to light novel aspects as the intensity of their impact.

Further search of *Drosophila* related literature provided useful information on insect response to environmental stressful conditions: it is suggested that adult insects can enter a state of reproductive dormancy under stressful conditions characterised by reduced metabolism and arrested oogenesis among other phenotypic expressions [37]. In *Drosophila* species reproductive arrest is controlled by juvenile hormone; a hormone that regulates all aspects of insect reproduction [27], [38]–[41]. In male Drosophila, the role effect of juvenile hormone is poorly understood [8], [39], [42]. It may not affect spermatogenesis but trigger protein synthesis in its accessory gland [40]. In females, this hormone is the regulating factor of reproduction activity and oocyte maturation [26]–[28], [31], [43], [44]. Therefore, the radiation as a stressful agent may affect the juvenile hormone system and arrest oogenesis.

However, the oogenesis arrest can not be exclusively explained by the down-regulation of this hormone. Warming insects –high temperature is a common default in this kind of studies- may produce ecdysteroids a group of closely-related steroid hormones secreted by prothoracic glands, which are located in the prothorax of the insect. These hormones trigger a cascade of physiological events that culminate in molting. Prothoracic glands produce and release ecdysteroids only after they have been stimulated by another chemical messenger, prothoracico-tropic hormone a peptide hormone secreted by the corpora cardiaca located on the aorta wall just behind the brain. The corpora cardiaca release their store of prothoracico-tropic hormone only after they receive a signal from neurosecretory cells in the brain. In a sense, they act as signal amplifiers sending out a big pulse of hormone to the body in response to a small message from the brain, controlling the egg-laying of the insect. We assume that this triggering needed small message may be the mobile phone related radiation. This mechanism is of value because it could be a potent explanatory pathway to surpass the conflict over the thermal or non thermal consequences of radiation. The same explanation has been used to explain the oocyte arrest [15]. Although our suggested biochemical explanation needs to be proven experimentally in the future, this may be complementary to previously published biophysical mechanisms which report magnetic alteration in cell membrane energy [45], alteration of the hydrophilic and hydrophobic properties of cell membrane [1], alterations of cell membrane ion channels [6], increasing oxidative stress in cell and animal models [8], [9], [26], [36], [46] after exposure to electromagnetic fields. We, further, believe that the MsrA induction -which is the major ecdysone induced protein in Drosophila- [47], [48] might be responsible for the inhibition of the transient oxidative stress action expressed by reactive oxygen species. Of note is that the insect ecdysteroid phosphate phosphatase is homologous to the human Sts-1 gene (HGNC: UBASH3B or ubiquitin associated SH3 domain containing B) which is a key regulator of T-cell activity [49].

In that prospect our explanation does not negate modern technology but illuminates pathways and protective mechanisms. Because *Drosophila* model genome is at least 54% homologous to the human, our findings may be of clinical value in terms of limit setting for public health safety.

We conclude that, a. the 1880 MHz radiation frequency may be a threshold frequency under which possible health effects on reproduction ability may appear, as the modified by Lipsey and Wilson g measure-based on Hedge's g bias correction for small samples, confirms. b. The near field of the mobile phone 900 MHz is stronger than the near field of the 1880 MHz (Fig 2, 3, 4) c. The electromagnetic powers of the two near fields become uneven and increase as h and radius decrease (Fig 2, 3, 4) d. SAR can neither be instrumentally measured not even indicatively given the small insects size nor accurately calculated in such a vicinity to the antenna e. The effect of the 900 MHz near field exposure is more intense than the effect of the 1880 MHz near field exposure.

The information could now correct assumptions in experimental designs and analysing concepts. The information could also correct assumptions about the impact and establish new criteria about the electromagnetic exposure safety of the continually and additively exposed user's population.
