# Correction: Diverse radiofrequency sensitivity and radiofrequency effects of mobile or cordless phone near fields exposure in *Drosophila melanogaster*

**DOI:** 10.1371/journal.pone.0225304

**Published:** 2019-11-12

**Authors:** Styliani Geronikolou, Stelios Zimeras, Constantinos H. Davos, Ioannis Michalopoulos, Stephanos Tsitomeneas

There is a corrigendum in reference 18. The correct reference is: Panagopoulos DJ, Karabarbounis A, and Margaritis LH (2004) Effect of GSM 900 MHz Mobile Phone Radiation on the Reproductive Capacity of *Drosophila melanogaster*. Electromagn Biol Med 23: 29–43.

In addition, there is a corrigendum in the reported temperature at which the glass vials containing food used for *D*. *melanogaster* experiments were stored. The penultimate sentence of the Food and culture subsection of the Materials and Methods should read: Finally, all the glass vials with food were kept at 4°C.
